# Surgical and Histopathologic Insights Into Mid‐Aortic Syndrome: A Case Report

**DOI:** 10.1155/cris/3337193

**Published:** 2026-08-29

**Authors:** Bernhard Hruschka, Panagiotis Doukas, Alexander Gombert

**Affiliations:** ^1^ Department for Vascular Surgery, RWTH University Hospital Aachen, Aachen, Germany, ukaachen.de

**Keywords:** abdominal aortic coarctation, case report, histopathology, mid-aortic syndrome, open surgical repair, pediatric hypertension

## Abstract

Mid‐aortic syndrome (MAS) is a rare condition marked by narrowing of the thoraco‐abdominal aorta, often causing severe hypertension. We present a 17‐year‐old male with refractory hypertension due to high‐grade aortic coarctation at the viscero‐renal segment. After failed endovascular interventions, open thoracoabdominal aortic repair was performed. The celiac trunk, superior mesenteric artery (SMA), and right renal artery were initially implanted into the tubegraft as a patch, but limited blood flow required revision including separate bypasses for the visceral arteries and both renal arteries. The patient was discharged with normalized blood pressure. Histopathology revealed adventitial necrosis, smooth muscle disorganization, and severe intimal thickening, suggesting intimal hyperplasia and fibrosis reduced blood flow. Separate bypasses might have been more effective than a patch for long‐term patency. At a 4‐year follow‐up, the patient remains stable, though the right renal bypass is occluded. This case report provides unique insights into the histopathological features of MAS and their potential impact on surgical decision‐making, contributing to our understanding of this rare condition.

## 1. Introduction

We present the case of a 17‐year‐old patient with a severe case of mid‐aortic syndrome (MAS) treated at the University Hospital RWTH Aachen, Department for Vascular Surgery. The patient and a parent, given the patient’s age at the time of operation, provided full written informed consent for the publication of this case report. MAS is a rare disease involving narrowing of the abdominal and/or distal thoracic aorta, often affecting the renal and visceral branches. First described by Sen et al. [1], MAS accounts for 0.5%–2% of aortic coarctation cases [2].

While antihypertensive medication is a crucial component of MAS management, it mainly addresses the symptoms rather than the underlying anatomical obstruction. Most patients require additional interventions, such as endovascular or open surgical repair, to achieve acceptable blood pressure control, and even after successful revascularization, many continue to need antihypertensive therapy. This underscores the complex nature of blood pressure management in MAS patients, where a multimodal treatment approach is often necessary [3–5].

The few existing studies on histopathology have documented adventitial necrosis, disorganization and aggregation of smooth muscle cells (SMCs) in the media, and significant disruption of elastic fibers [2, 3, 6].

This report illustrates the critical role of vessel wall pathology in determining surgical success, offering valuable guidance for reconstruction strategies in challenging MAS cases.

## 2. Report

**Figure 1:**
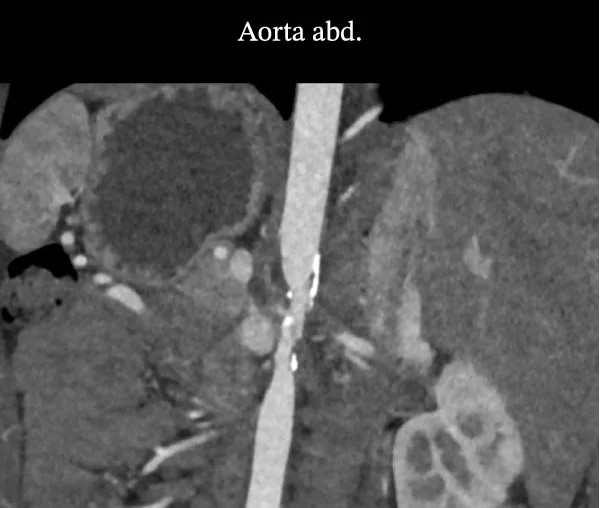
CT angiography, coronary (posterior–anterior) view—severe narrowing of the aorta in the juxtarenal segment.

**Figure 2:**
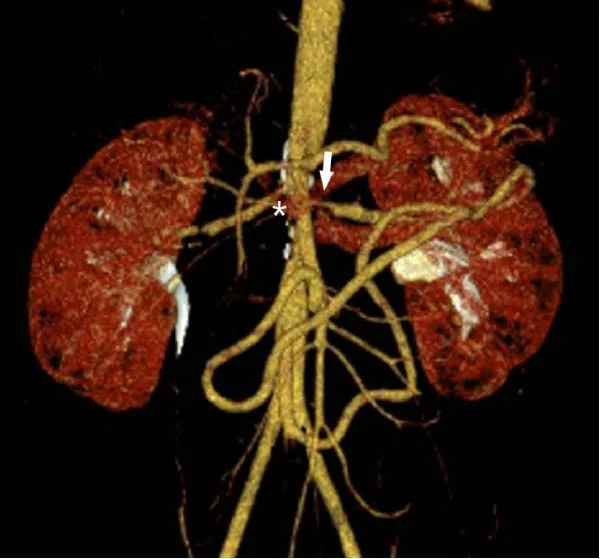
CT angiography, 3D‐reconstruction, ap view—both renal arteries showing signs of stenosis, left (arrow) is more pronounced than right (asterisk).

**Figure 3:**
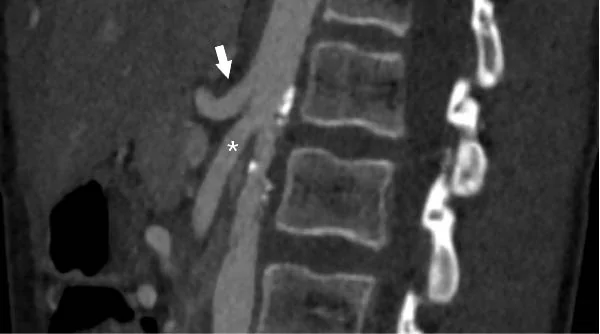
CT angiography, lateral view—neither celiac trunk (arrow) nor SMA (asterisk) showing visible signs of severe stenosis.

A 17‐year‐old patient presented to a pediatric cardiologist with severe hypertension and a tall stature (1.96 m, >97^th^ percentile), prompting further investigation. A computed tomography (CT) scan led to a diagnosis of MAS at an external hospital, with the primary stenosis located in the abdominal aorta below the celiac trunk and superior mesenteric artery (SMA), reducing the aortic diameter from 13 mm in healthy segments to 3 mm in the diseased segment, as seen in Figure 1. Both renal arteries also showed proximal stenosis, with the left renal artery being affected more severely (Figure 2). Neither the celiac trunk nor the SMA showed signs of severe stenosis, as visible in Figure 3.

On clinical examination, the patient was hypertensive, with a marked blood pressure differential between the upper and lower extremities. The femoral pulses were palpable but distinctly weaker than the radial pulses. No abdominal or flank bruits were audible. Apart from the tall stature (1.96 m, >9^7th^ percentile), no further dysmorphic or syndromic features were noted.

After conservative medical therapy including metoprolol, amlodipine, and candesartan, failed to lower blood pressure, endovascular options were explored. The patient underwent three angioplasties of the aorta and both renal arteries over the course of 24 months at an external hospital, initially reducing the pressure gradient from 50 to 30 mmHg, but hypertension remained uncontrolled.

The patient was referred to our institution by a pediatric cardiologist to discuss open aortic reconstruction. The decision for open surgical repair was primarily driven by concerns about potential complete aortic occlusion and the possibility of improved blood pressure control. All parties agreed on an open approach. At referral to our institution, the directly preoperative blood pressure was 139/87 mmHg in the upper extremity and 112/70 mmHg in the lower extremity.

As part of the preoperative workup, echocardiography demonstrated a normal left ventricular function with an ejection fraction of 72%. Pulmonary function testing revealed age‐appropriate lung capacity and FEV1. All blood levels showed age‐appropriate values within the normal ranges. Bilateral carotid artery duplex ultrasonography yielded unremarkable findings. Comprehensive anesthesiological and internal medicine evaluations were unremarkable except for arterial hypertension. Echocardiography demonstrated good biventricular function (ejection fraction 72% and shortening fraction 42%) without hypertrophy, a physiological pulmonary valve regurgitation, no pericardial effusion, and a normal aortic root diameter of 27 mm, arguing against a Marfan‐type connective tissue disorder. Secondary and inflammatory causes were considered and excluded: inflammatory markers were within normal limits (C‐reactive protein <0.3 mg/L and leucocytes 6.7/nL), renal and hepatic parameters were normal, and there were no constitutional symptoms.

**Figure 4:**
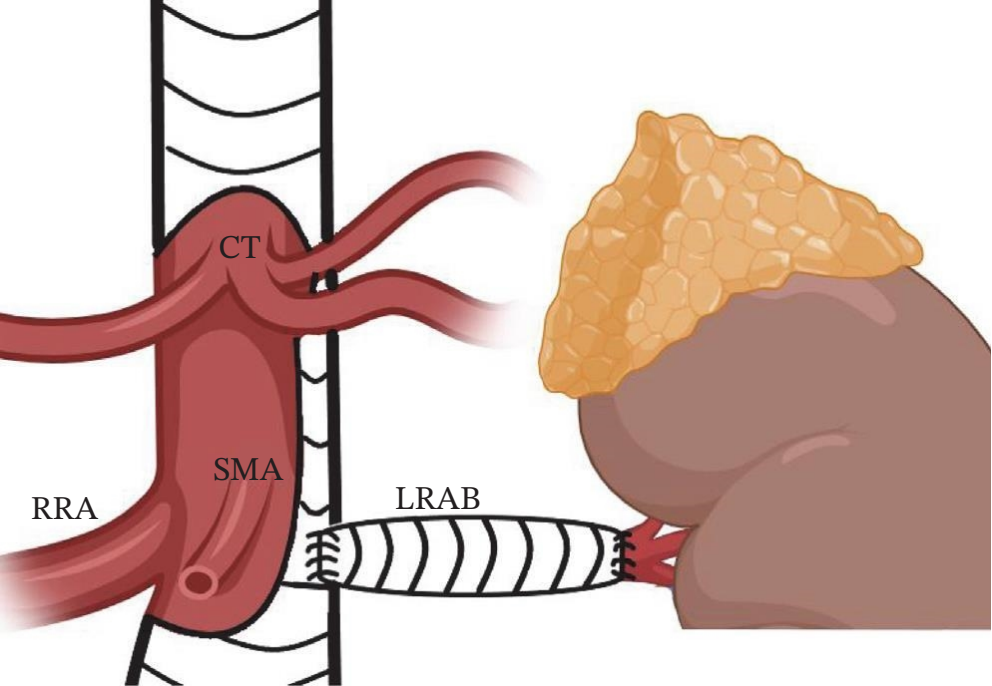
Schematical drawing of the primary reconstruction using an island patch including the celiac trunk (CT), the right renal artery (RRA), and the left renal artery bypass (LRAB). The figure is purely schematic and does not reflect exact anatomical relationships.

Both operations were performed through a thoracoabdominal approach. The initial open surgical reconstruction was performed as a type IV thoracoabdominal aortic repair using cardiopulmonary bypass. The procedure involved implanting the celiac trunk, SMA, and right renal artery as a common patch (island) into a 14 mm tube graft, with a separate 8 mm‐bypass to the left renal artery. The right renal artery was included in the island patch because of its close anatomical proximity to the celiac trunk and SMA, whereas the more severely diseased and separately located left renal artery was revascularised by a dedicated bypass. Intraoperative anticoagulation was achieved with heparin at a dose of 3 mg/kg under continuous activated clotting time (ACT) monitoring, aiming for an ACT of 200–220 s. At the end of the procedure, the excessive effect of heparin was reversed with protamine, also under ACT monitoring. Postoperatively, the patient was admitted to the intensive care unit (ICU). A schematic drawing of the primary reconstruction can be seen in Figure 4.

**Figure 5:**
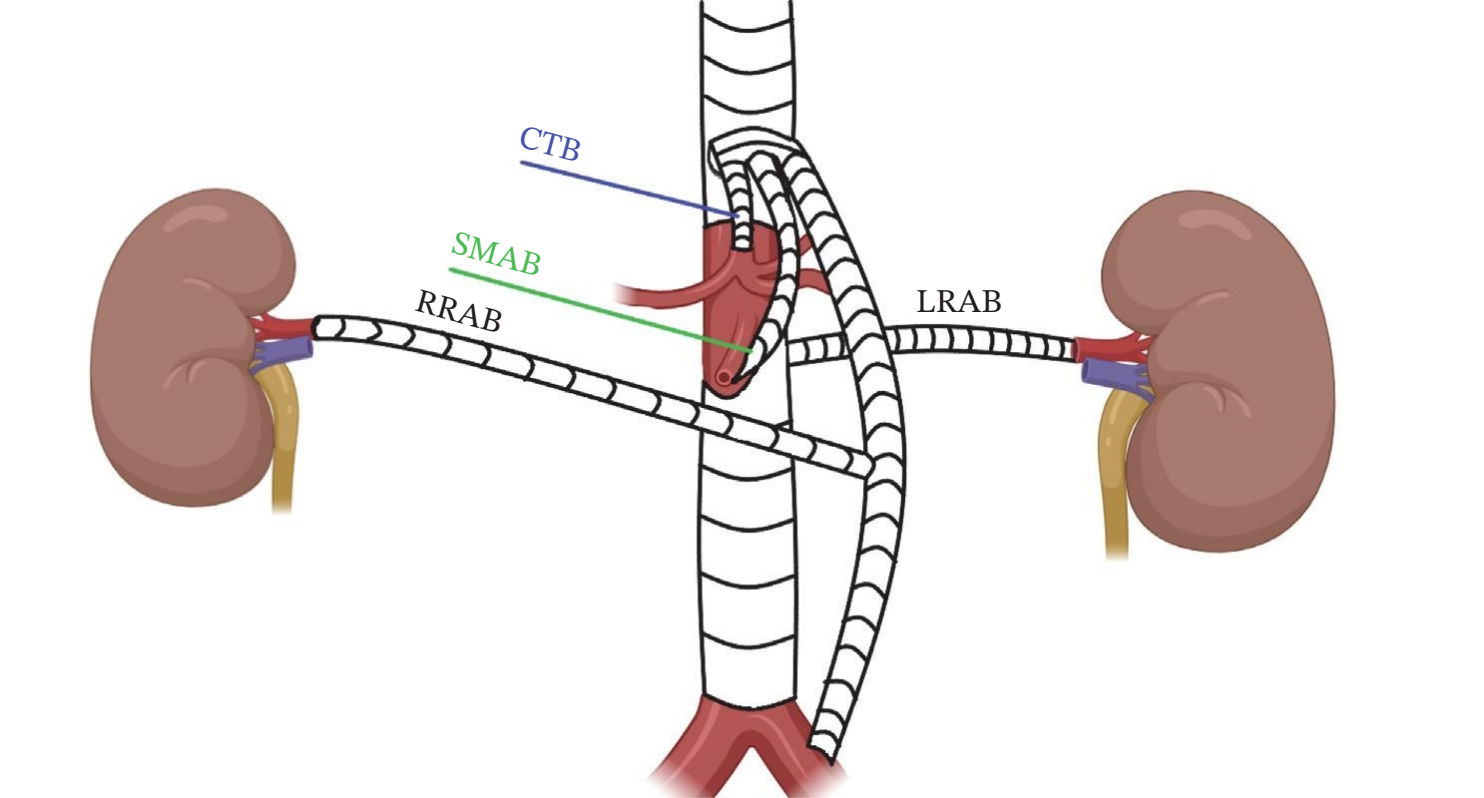
Schematic representation of the reconstruction after revision. It illustrates the Y‐graft anastomosed to the supraceliac aorta together with a 10‐mm jump bypass to the left common iliac artery. One limb of the Y‐graft provides an end‐to‐end celiac trunk bypass (CTB, blue), and the other limb an end‐to‐side superior mesenteric artery bypass (SMAB, green). The right renal artery bypass (RRAB) receives its inflow from the jump bypass to the left iliac artery. The figure is purely schematic and does not reflect exact anatomical relationships.

**Figure 6:**
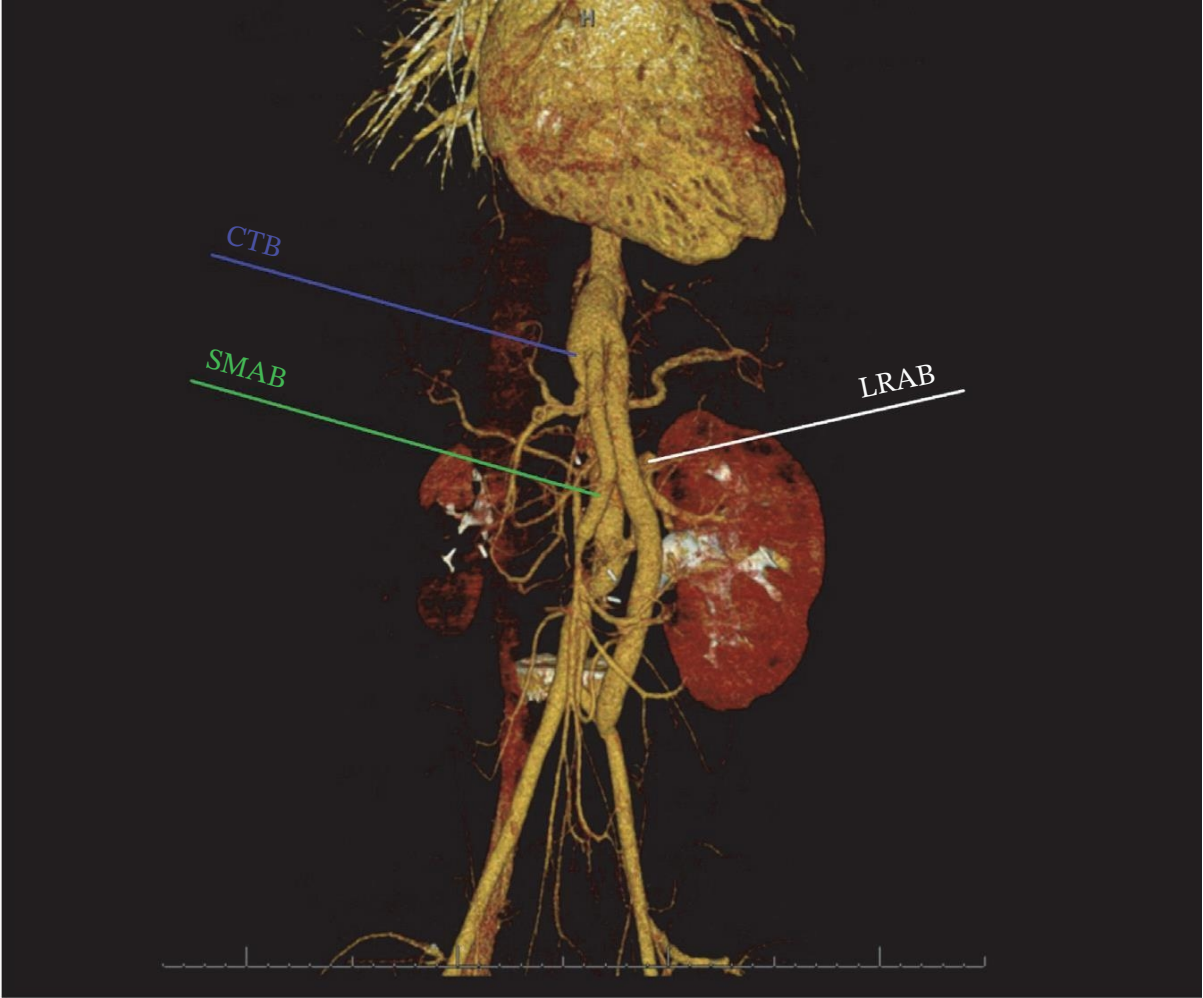
3D‐reconstruction of the CT angiography of the definitive reconstruction at follow‐up after six months. The Y‐graft arises from the supraceliac aorta. Its limbs supply the celiac trunk (end‐to‐end, CTB) and the SMA (end‐to‐side onto the lateral wall of the more cranial SMA segment, SMAB). The 6 mm graft to the right renal artery originates from the iliac jump‐graft, which, in this CT is already occluded and thus not visible. The bypass for the left renal artery (LRAB) remained unchanged.

Three days later, due to progressive nausea and an episode of fresh blood hematemesis, the patient underwent emergency esophagogastroduodenoscopy, which revealed an actively bleeding ulcer together with ischemia of the greater curvature of the stomach. The clinical picture was accompanied by markedly elevated transaminases (AST 1860 U/L and ALT 1978 U/L) and a rising serum lactate (up to 5.3 mmol/L). Owing to this suspected gastric ischemia, CT imaging revealed patent but poorly perfused vessels through the initial patch reconstruction, namely, the right renal artery, SMA, and celiac trunk, necessitating surgical revision. The separate bypass to the left kidney was still patent and well‐perfused. After re‐thoracophrenolaparotomy reopening the same exposure, the tube‐part of a Y‐graft was anastomosed end‐to‐side to the healthy supraceliac aorta. The two 7 mm limbs of the Y‐graft were connected to the celiac trunk and the SMA, respectively—the celiac trunk limb in an end‐to‐end fashion and the SMA limb in an end‐to‐side fashion onto the lateral wall of the more cranial segment of the SMA (as depicted in Figures 5 and 6). A separate 10 mm bypass from the Y‐graft to the left common iliac artery was created. Because the native distal aorto‐iliac segment remained diffusely diseased, including a narrowed left common iliac artery (Figure 2), this jump bypass was intended to secure antegrade perfusion of the pelvis and lower extremities across the previously coarcted segment; it also provided a healthy, disease‐free inflow source for renal revascularisation, from which a 6 mm graft was anastomosed end‐to‐end to the right renal artery, this origin being chosen for the most favorable, tension‐free course to the caudally located right renal artery.

Post‐surgery, blood flow to all organs was satisfactory, and the patient was again admitted to ICU. Early postoperative CT angiography on day five demonstrated the patency of all bypass grafts. Postoperative blood pressure control was achieved with reduced doses of amlodipine and metoprolol. Initial anticoagulation consisted of Heparin under continuous aPTT monitoring, followed by transition to Rivaroxaban for a 12‐month course.

After 47 days of postoperative in‐hospital stay, the patient was discharged in good general condition with normalized blood pressure levels and reduced need for antihypertensive medication. Long‐term surveillance consists of biannual cardiologic evaluations and annual follow‐up visits at our vascular surgery outpatient clinic. At a 4‐year follow‐up, the patient remains in good health. The right renal bypass followed a distinct course: it was patent on the early postoperative CT angiography on day five; routine CT imaging at six months raised the suspicion of occlusion, but subsequent magnetic resonance imaging (MRI) and CT angiography demonstrated a severe stenosis rather than complete occlusion; at the 4‐year follow‐up, CT angiography showed the graft to be occluded, while all other vessels and bypasses remained patent. The exact timing and mechanism of this progression remain unclear. The occlusion has remained clinically silent, with renal function preserved at an eGFR of 111.8 mL/min, indicating adequate compensation by the left kidney. While the patient still requires antihypertensive medication, including candesartan and empagliflozin, the level of antihypertensive medication has been reduced compared to preoperative levels and is currently managed by the patient’s cardiologist. The preoperative trans‐stenotic gradient of 50 mmHg (reduced only to 30 mmHg by repeated angioplasty) and the preoperative upper‐versus‐lower‐extremity differential (139/87 vs. 112/70 mmHg) were abolished after definitive separate bypass reconstruction, with an upper‐extremity pressure of 122/79 mmHg after the index operation and 130/80 mmHg after the revision; at four years, the patient records self‐measured systolic values of 120–140 mmHg with no detectable gradient. The definitive reconstruction at six months is shown in Figure 6.

Intraoperatively, we collected a 1.2 cm × 1.0 cm segment of the diseased abdominal aorta, stored it in formaldehyde, and performed histopathological analysis after staining with hematoxylin and eosin (HE), picrosirius‐red, Elastica‐van‐Giesson (EvG), and Movat–Verhöff.

**Figure 7:**
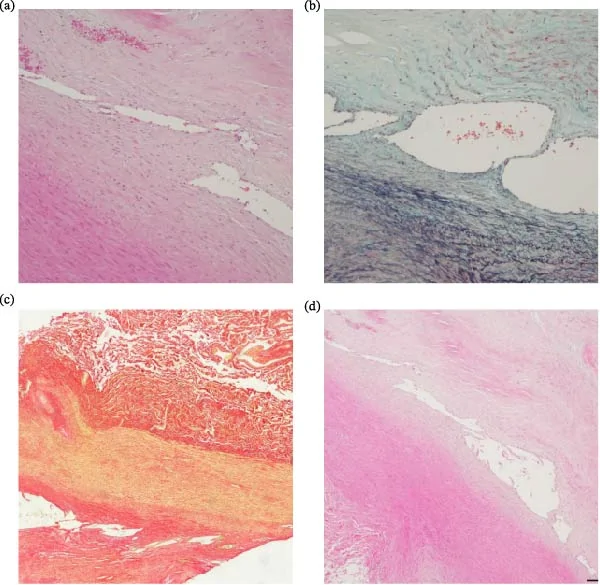
Overview of histopathologic findings in different stainings. (a) HE: multiple relatively thin‐walled large vessels at the media towards the luminal surface with mild neutrophil aggregations around the vessels (magnification ×40). (b) Movat–Verhöff: moderate to severe degenerative changes of elastic fibers and with its complete loss towards the luminal surface. The remaining part showed partial loss, fragmentation, thinning, clump formation, and loss of its wavy appearance (magnification ×40). (c) Picrosirius red: media with abundant necrotic changes together with disruption of the connective tissue and SMC arrangement, degenerative changes (magnification ×20). (d) HE: large area of acute necrosis of the connective tissue of coagulative type (most likely ischemic), together with widespread, recent, and old hemorrhages and hemolysis (magnification ×40).

HE staining showed extensive ischemic necrosis, hemorrhages, and hemolysis in the adventitia. The media had significant necrosis, connective tissue disruption, and severe SMC disarrangement, with an unusually large vasa vasorum. The intima displayed severe thickening with degenerative changes. Movat–Verhöff staining indicated severe elastic fiber degeneration, while EvG staining revealed severe fibrous connective tissue changes across all layers. An overview of the pathologic changes can be seen in Figure 7.

## 3. Discussion

At age 15 at the time of diagnosis, two years before open surgery, our patient’s presentation was relatively late as MAS typically becomes clinically apparent in infancy or early childhood [3, 7]. Current evidence strongly supports open surgical repair over endovascular approaches. Long‐term data demonstrate that freedom from reintervention after endovascular procedures is 58% at 1 year and 33% at 5 years, while open surgical procedures achieve superior outcomes with 83% freedom from reintervention at 1 year and 72% at 10 years [3, 4]. Although endovascular methods like balloon dilatation or stenting offer less invasive options, they are associated with higher restenosis rates and are less suitable for complex cases. Open surgical techniques, including aorto‐aortic bypass grafting, interposition bypass, and patch angioplasty, provide the best long‐term outcomes [1, 4, 6, 8–10]. An alternative strategy to the one used in this case—leaving the coarcted aorta in situ and using extra‐anatomic bypass grafts for both the aorta and its branches—was considered. We preferred in‐line anatomical reconstruction for its superior long‐term durability, especially in a young patient.

Patient age significantly influences the choice of the intervention strategy. Cortenbach et al. [10] demonstrated that patients under 18 years of age showed better blood pressure control at follow‐up after open surgical repair compared to endovascular treatment. Open repair with patch angioplasty is particularly advantageous in pediatric patients as it allows for vessel growth, unlike endovascular stenting, which may require future revision due to patient growth [9–11]. When evaluating potential treatment strategies, the underlying characteristics of the lesion should be carefully considered, as the extent of vessel wall changes can influence surgical technique selection and potentially affect long‐term outcomes. The histopathologic findings in this case reveal severe fibrotic processes and endothelial hyperplasia, consistent with the observations reported in the literature [6]. These changes likely occur as a secondary reaction to wall stress. Notably, enlarged and dilated vasa vasorum were observed on the luminal side of the media, potentially resulting from hypoxia‐induced neoangiogenesis triggered by vessel stenosis and intima hyperplasia [6]. Such vascular adaptations resemble those seen in severely arteriosclerotic vessels [6]. In this case, severe degenerative changes like fragmentation, loss, thinning, and clumping of elastic fibers were observed, in accordance with the findings by Heider et al. [2] Collagenous fibers exhibited a sparse distribution and appeared coalescent, with a marked disturbance in fiber arrangement compared to healthy vessels. The etiology of MAS remains mostly unclear, with most cases classified as idiopathic [1]. Poulias et al. [6] described “advanced fibrotic proliferation of the media,” which aligns with our EvG staining results. Endothelial thickening, a predominant finding reported by Poulias et al. [6] and Hallett et al. [5], was also observed in our case. More recently, Heider et al. [2] presented a case series of eight pediatric patients with abdominal aortic coarctation and hypoplasia, who consistently showed intimal hyperplasia, with disorganization of elastic fibers and focal medial necrosis in some cases. These findings generally correlate with our results.

The observed severe intimal hyperplasia and fibrosis of the aorta, particularly at the level of visceral and renal vessel ostia, corresponded with the inadequate perfusion seen clinically after the initial patch reconstruction. Preoperative CT imaging demonstrated stenosis of both renal arteries, considerably more pronounced on the left side, while the celiac trunk and SMA appeared unstenosed. The close anatomical proximity of the celiac trunk, SMA, and right renal artery initially suggested suitability for an island patch reconstruction. However, the post‐operative course revealed this approach to be insufficient. By employing separate bypasses for celiac trunk, SMA and both renal arteries, respectively, in the first place, a revision may have been avoided. While our histopathological findings might suggest a correlation with surgical outcomes, other factors such as technical considerations, hemodynamics, and individual patient characteristics may have also contributed to the initial reconstruction failure.

The tall stature (1.96 m) was the only feature raising the question of an underlying connective tissue disorder; no other syndromic features (e.g., of Marfan, Loeys‐Dietz, Williams, Alagille, or neurofibromatosis type 1) were present clinically. No genetic testing was performed prior to the operation, and none has been undertaken during follow‐up. In retrospect, formal genetic and rheumatologic evaluation would have been desirable to definitively exclude a heritable or inflammatory etiology.

The histopathological pattern in our case is distinct from the principal differential diagnoses of long‐segment abdominal aortic narrowing. In fibromuscular dysplasia, the dominant abnormality is medial fibroplasia producing the characteristic alternating webs and microaneurysms (the “string‐of‐beads” appearance), typically without the transmural intimal hyperplasia or the adventitial ischemic necrosis, hemorrhage, and disorganized media that we observed [12]. Takayasu arteritis, the most important inflammatory mimic, is characterized by granulomatous transmural inflammation with giant cells and adventitial thickening; the absence of any inflammatory infiltrate, together with normal inflammatory markers and the lack of constitutional symptoms, argues firmly against a vasculitic etiology [13]. The findings are instead most consistent with the dysplastic, fibrotic and intimal‐hyperplastic process described in idiopathic MAS. A limitation of our pathological assessment is that tissue was obtained only from the aortic wall; the ostial lesions of the visceral and renal branches were not separately harvested. We hypothesize that the branch‐ostial stenoses reflect the same continuous intimal‐hyperplastic and fibrotic process as the aortic wall rather than a separate mechanism, which would also explain why incorporating the diseased native ostia within the island patch failed to relieve the obstruction. The mechanism of occlusion of the right renal bypass remains unclear. Suboptimal graft geometry and length might have promoted thrombosis in the conduit; an alternative explanation is hemodynamic fluctuations with consequent low‐flow. In addition, because the right renal graft originated from the iliac jump‐bypass rather than directly from the aortic reconstruction, competitive flow within this parallel pelvic pathway may have reduced flow velocity in the renal conduit and predisposed it to eventual occlusion. Mechanical kinking is unlikely due to the images gathered in the post‐revision CT.

Because the aortic wall carried severe intimal hyperplasia and fibrosis demonstrated histologically, the ostia remained flow‐limiting even after the new conduit was in place. The deficiency was therefore likely not a technical failure of the anastomoses but the inclusion of diseased native ostial tissue within the patch, which was initially deemed suitable. This also accounts for the need to bypass the celiac trunk and SMA at revision, although they had not appeared stenotic on the preoperative imaging. On day 3 CT, these vessels were patent within the patch yet hypoperfused, confirming that the patch construct itself was flow‐limiting and prompting conversion to separate end‐to‐end and end‐to‐side bypasses.

MAS represents a significant, yet underreported cause of secondary hypertension in children and young adults requiring careful diagnostic evaluation. Clinical management of MAS requires an individualized therapeutic approach, combining antihypertensive medication with carefully selected strategies, such as endovascular interventions or open surgical repair, tailored to each patient’s specific anatomical and clinical characteristics. The histopathologic analysis showed mostly fibrotic changes affecting the aortic wall of the stenotic segment. This case report demonstrates a possible correlation between specific histopathological findings and surgical outcomes in MAS, suggesting that severe intimal hyperplasia and fibrosis may favor separate bypass grafting over patch reconstruction in complex cases requiring visceral and renal revascularization. Further research would be valuable to validate this approach.

## Funding

No funding was received for this study. Open Access funding enabled and organized by Projekt DEAL.

## Consent

The patient and a parent, given the patient’s age at the time of operation, provided full written informed consent for the publication of this case report.

## Conflicts of Interest

The authors declare no conflicts of interest.

## Data Availability

The data that support the findings of this study are available from the corresponding author upon reasonable request.
